# Independent effect of Aβ burden on cognitive impairment in patients with small subcortical infarction

**DOI:** 10.1186/s13195-023-01307-5

**Published:** 2023-10-14

**Authors:** Sung Hoon Kang, Minwoong Kang, Jung Hoon Han, Eun Seong Lee, Keon-Joo Lee, Su Jin Chung, Sang-Il Suh, Seong-Beom Koh, Jae Seon Eo, Chi Kyung Kim, Kyungmi Oh

**Affiliations:** 1 Department of Neurology, Korea University Guro Hospital, Korea University College of Medicine, 148 Gurodong-ro, Guro-gu, Seoul, 08308 South Korea; 2 Department of Biomedical Research Center, Korea University Guro Hospital, Korea University College of Medicine, Seoul, South Korea; 3 Department of Nuclear Medicine, Korea University Guro Hospital, Korea University College of Medicine, 148 Gurodong-ro, Guro-gu, Seoul, 08308 South Korea; 4 Department of Neurology, Myongji Hospital, Hanyang University College of Medicine, Goyang, South Korea; 5 Department of Radiology, Korea University Guro Hospital, Korea University College of Medicine, Seoul, South Korea

**Keywords:** Amyloid-β (Aβ), Cognitive trajectories, Post-stroke cognitive impairment (PSCI), Small subcortical infarction

## Abstract

**Background:**

The effect of amyloid-β (Aβ) on cognitive impairment in patients with small subcortical infarction remains controversial, although a growing body of evidence shows a substantial overlap between Alzheimer’s disease (AD) and subcortical ischemic vascular dementia, another form of cerebral small vessel disease (cSVD). Therefore, we investigated the relationships between Aβ positivity and the development of post-stroke cognitive impairment (PSCI) in patients with small subcortical infarction.

**Methods:**

We prospectively recruited 37 patients aged ≥ 50 years, with first-ever small subcortical infarction, who underwent amyloid positron emission tomography, 3 months after stroke at Korea University Guro Hospital. We also enrolled CU participants matched for age and sex with stroke patients for comparison of Aβ positivity. Patients were followed up at 3 and 12 months after the stroke to assess cognitive decline. Logistic and linear mixed-effect regression analyses were performed to identify the effect of Aβ positivity on PSCI development and long-term cognitive trajectories.

**Results:**

At 3 months after stroke, 12/37 (32.4%) patients developed PSCI, and 11/37 (29.7%) patients had Aβ deposition. Aβ positivity (odds ratio [OR] = 72.2, *p* = 0.024) was predictive of PSCI development regardless of cSVD burden. Aβ positivity (*β* = 0.846, *p* = 0.014) was also associated with poor cognitive trajectory, assessed by the Clinical Dementia Rating-Sum of Box, for 1 year after stroke.

**Conclusions:**

Our findings highlight that Aβ positivity is an important predictor for PSCI development and cognitive decline over 1 year. Furthermore, our results provide evidence that anti-AD medications may be a strategy for preventing cognitive decline in patients with small subcortical infarctions.

## Background

A considerable number of post-stroke survivors suffer from post-stroke cognitive impairment (PSCI), which in turn leads to impaired activities of daily living and an increased burden on caregivers, regardless of physical disability [1–3]. Alzheimer’s disease (AD), characterized by the deposition of amyloid-β (Aβ) in the brain is the most common cause of dementia, and AD pathology may be an important risk factor for the development of PSCI. Animal studies have shown that cerebral ischemia triggers accelerated Aβ deposition [4]. Despite the apparent association in animal studies, the interaction between PSCI and Aβ deposition in humans remains controversial.

Cerebral small vessel disease (cSVD) causes various clinical syndromes, including subcortical ischemic vascular dementia (SIVD) and lacunar syndromes. Different from PSCI, a growing body of evidence shows that AD and SIVD affect one another interactively [5], and Aβ deposition and cSVD burden have synergistic effects on cognitive decline. The mechanism of PSCI may differ from by ischemic stroke subtype (territorial, lacunar, or cardioembolic infarction). However, many previous studies investigated the Aβ positivity in all types of stroke survivors and, therefore, failed to focus on one etiology of ischemic stroke. The effects of lesion size and strategic location may override the role of Aβ deposition in patients with PSCI with territorial and strategic infarctions.

Therefore, in this study, we investigated the relationship between Aβ deposition and development of PSCI. Additionally, we explored the cognitive trajectory after ischemic stroke based on Aβ positivity. To minimize the effect of stroke lesion size and strategic site, participants were limited to those with small subcortical infarctions. Considering the substantial overlap between AD and SIVD, we hypothesized that Aβ deposition is predictive of the development of PSCI in patients with small subcortical infarctions.

## Methods

### Study participants

Patients, aged ≥ 50 years with first-ever ischemic stroke, who were admitted to Korea University Guro Hospital within 7 days of ischemic stroke were screened for enrollment eligibility from June 1, 2021, to April 1, 2022. At baseline, all patients with ischemic stroke underwent a comprehensive stroke evaluation, including neurological examination using the National Institutes of Health Stroke Scale (NIHSS), functional outcome using the modified Rankin scale, and brain magnetic resonance imaging (MRI).

We prospectively enrolled patients with small subcortical infarctions to eliminate the effects of the stroke lesion size and strategic site. We excluded patients with the following conditions: (1) strategic infarcts involving the anterior thalamus and hippocampus; (2) severe aphasia (NIHSS language score > 1), visual impairment, or physical disabilities (Modified Rankin Scale score > 2) due to ischemic stroke; and (3) presence of premorbid cognitive impairment, neurodegenerative diseases, lobar hemorrhage, or psychiatric disorders.

Premorbid cognitive impairment was determined using the Informant Questionnaire on Cognitive Decline in the Elderly (IQCODE) [6], answered by the participants’ spouses or a first-degree relative. A score of ≥ 3.6 on the IQCODE indicated premorbid cognitive impairment, and the participants were excluded [6].

Hypertension was defined as a diagnostic history of hypertension or current use of any antihypertensive medication, and diabetes was defined as a diagnostic history of diabetes or current use of any anti-diabetic medication.

### Cognitively unimpaired (CU) participants without stroke

We also enrolled CU participants matched for age and sex with stroke patients. These participants did not have a history of stroke, neurodegenerative disease, or psychiatric disorders and were composed of spouses of patients who visited the neurology clinic, volunteers who applied for comprehensive dementia evaluation advertised in the paper, and participants who had cognitive complaints. They visited the Memory Clinic in the Department of Neurology at Korea University Guro Hospital and underwent a comprehensive dementia evaluation.

All CU participants met the following criteria: (1) no medical history which is likely to affect cognitive function based on Christensen’s health screening criteria [7], (2) no objective cognitive impairment from comprehensive neuropsychological test battery on any cognitive domains (no cognitive test fell more than 1.0 standard deviation [SD] below age-adjusted norms), (3) independent in activities of daily living, and (4) neither structural lesions nor severe white matter hyperintensities (WMH) on brain MRI.

Hypertension was defined as a diagnostic history of hypertension or current use of any antihypertensive medication, and diabetes was defined as a diagnostic history of diabetes or current use of any anti-diabetic medication.

This study was approved by the Institutional Review Board of the Korea University Guro Hospital. Written informed consent was obtained from all participants.

### Follow-up assessment

All patients with stroke were followed up at 3 and 12 months after ischemic stroke. At the follow-up visit at 3 months, the patients underwent a neuropsychological battery using the Korean version of the Vascular Cognitive Impairment Harmonization Standards neuropsychological battery (K-VCIHS-NP) [8], Korean version of the Mini-Mental Status Examination (K-MMSE) [9], Clinical Dementia Rating-Sum of Box (CDR-SOB) [10], amyloid PET, and a second MRI. At the follow-up visit at 12 months, patients underwent the K-MMSE and CDR-SOB to evaluate the trajectory of global cognition.

CU participants without stroke were also followed up at 12 months after the initial comprehensive evaluation. At the follow-up visit at 12 months, CU participants also underwent the K-MMSE and CDR-SOB.

### Comprehensive neuropsychological battery

Patients underwent neuropsychological testing using the K-VCIHS-NP [8]. Seven cognitive measures were included in the battery, which were representative and important neuropsychological tests to evaluate the cognitive function in five cognitive domains as follows: (1) memory: the Seoul Verbal Learning Test (SVLT) delayed recall (verbal memory); (2) language: Korean version of the Boston Naming Test (K-BNT); (3) visuospatial function: the Rey Complex Figure Test (RCFT) Copying Test; and (4) frontal-executive function: the Digit Span Test Backward, animal component of the Controlled Oral Word Association Test (COWAT), and phonemic component of the COWAT and the Stroop Test (color reading). Results with continuous numeric values were converted to *z*-scores using the age, sex, and education criteria presented in the K-VCIHS-NP, and the *z*-scores were used in the analysis.

### Diagnosis of vascular cognitive impairment and vascular dementia

PSCI was defined according to the modified Peterson criteria and results of the K-VCIHS-NP. In the K-VCIHS-NP, cognitive functions were classified as impaired when objective cognitive impairment was − 1.5 SD on at least two different cognitive domains. The frontal domain consists of four neuropsychological tests. Cognitive impairment in the frontal domain was classified as objective cognitive impairment − 1.5 SD on two or more tests.

### Amyloid PET acquisition and visual reading

The patients underwent ^18^F-florbetaben PET using a discovery MI PET/computed tomography (CT) scanner (GE Medical Systems, Milwaukee, WI, USA). A 20-min emission PET scan in dynamic mode (comprising 4 × 5 min frames) was performed 90 min after the injection of a mean dose of 296 MBq ^18^F-florbetaben. Three-dimensional PET images were reconstructed in a 384 × 384 matrix with 0.65 × 0.65 × 2·79 mm voxel size using the ordered-subsets expectation maximization algorithm (iteration = 8 and subset = 34).

Amyloid PET images were reviewed by three experienced physicians (one neurologist and two nuclear medicine doctors) who were blinded to clinical information and dichotomized as either Aβ positive or negative using visual reads [11]. ^18^F-florbetaben PET was classified as positive when interpreters scored the visual assessment as 2 or 3 on the brain amyloid-plaque load (BAPL) score [11, 12]. Specifically, the regional cortical tracer uptake (RCTU) score was used for four brain areas (lateral temporal cortex, frontal cortex, posterior cingulate, cortex/precuneus, and parietal cortex). RCTU scores of 1, 2, and 3 indicated no tracer uptake, moderate tracer uptake, and pronounced tracer uptake, respectively.

An RCTU score of 1 in each brain region corresponded to a BAPL score of 1, an RCTU score of 2 in any brain region and no score of 3 corresponded to a BAPL score of 2, and an RCTU score of 3 in any of the four brain regions corresponded to a BAPL score of 3. Inter-rater agreement was excellent (Fleiss, *k* = 0.89). After the physicians individually rated, we determined the final Aβ positivity based on the majority of the visual reading result.

### MRI acquisition and WMH visual rating

We acquired standardized three-dimensional T1 Turbo Field Echo and three-dimensional fluid-attenuated inversion recovery (FLAIR) images using a 3.0-T MRI scanner (Philips 3.0T Ingenia Elition X; Philips Healthcare, Andover, MA, USA) by following imaging parameters: sagittal slice thickness, 0.6 mm; no gap; TR, 4800 ms; TE, 363 ms; flip angle, 90°; and matrix size, 288 × 287 pixels. As described previously [13], the Clinical Research Center for Dementia of South Korea WHM visual rating scale was used to investigate WMH in the deep subcortical and periventricular regions of the FLAIR images.

Briefly, deep WMH (DWMH) were classified as D1 (< 10 mm), D2 (10–25 mm), or D3 (≥ 25 mm) based on the longest lesion diameter. Periventricular WMH (PWMH) were classified as P1 (cap and band < 5 mm), P2 (5–10 mm), or P3 (cap or band ≥ 10 mm) based on the maximum length measured perpendicular (cap) and horizontal (band) to the ventricle. The combination of these D and P ratings yielded nine cells and the overall WMH severity (minimal, moderate, and severe) was defined based on the following combinations of D and P ratings: minimal (D1P1 and D1P2), moderate (D1P3, D2P1, D2P2, D2P3, D3P1, and D3P2), and severe (D3P3).

We also counted the number of microbleeds (MBs), defined as ≤ 10 mm in diameter on 150 axial slices of T2 susceptibility-weighted imaging-MRI by following imaging parameters: sagittal slice thickness, 2.0 mm; no gap; TR, 24 ms; TE, 0 ms; flip angle, 18°; and matrix size, 384 × 383 pixels [14]. Strictly lobar MBs (number of lobar MBs ≥ 1 and deep MBs = 0) and cerebral superficial siderosis (cSS) were considered as cerebral amyloid angiopathy (CAA) markers. Regarding lobar MBs, the lobar regions were according to the criteria proposed by Gregoire et al. [15]. cSS was defined as chronic blood linear residues in superficial layers of the cerebral cortex [16].

### Statistical analyses

For comparison between the clinical characteristics of stroke and CU without stroke groups, independent *t*-tests and chi-squared tests were used. To assess the effect of stroke on longitudinal cognitive changes, we performed a linear mixed-effect regression analysis and included stroke group (stroke vs CU without stroke), time, and stroke group × time as fixed effects, along with age, sex, years of education, hypertension, diabetes, Aβ positivity, and WMH severity. The patients were included as random effects. To identify the association of stroke with cognitive function at 3 months and 12 months, we performed linear regression analyses with the stroke group as a predictor after controlling for with age, sex, years of education, hypertension, diabetes, Aβ positivity, and WMH severity.

In stroke groups, independent *t*-tests and chi-squared tests were used to compare the clinical characteristics of the post-stroke normal cognition (PSNC) and PSCI groups. To investigate the association between Aβ deposition and the development of PSCI, we performed a logistic regression analysis with Aβ positivity, hypertension, diabetes, and WMH severity as predictors and PSCI development as an outcome after controlling for age, sex, and years of education. To assess the effect of Aβ positivity on longitudinal cognitive changes, we performed a linear mixed-effect regression analysis and included Aβ positivity, time, and Aβ positivity × time as fixed effects, along with age, sex, years of education, hypertension, diabetes, and WMH severity. The patients were included as random effects. To identify the association of Aβ positivity with cognitive function at 3 months and 12 months, we performed linear regression analyses with Aβ positivity as a predictor after controlling for with age, sex, years of education, hypertension, diabetes, and WMH severity.

All reported *p*-values were two-sided, and the significance level was set at 0.05. All analyses were performed using R version 4.3.0 (Institute for Statistics and Mathematics, Vienna, Austria; http://www.r-project.org, RRID: SCR_001905).

## Results

### Clinical characteristics of the participants

Of the 52 patients with stroke prospectively enrolled for the baseline evaluation, 15 missed the cognitive assessment follow-up at 3 months. The mean age (*p* = 0.920), female ratio (*p* = 1.000), years of education (*p* = 0.196), and rates of diabetes (*p* = 1.000) did not differ between the patients with stroke and CU participants without stroke. Patients with stroke had a higher frequency of hypertension (73.0% vs 45.9%; *p* = 0.033) and WMH burdens (moderate WMH, 24.3% vs 5.4%; severe WMH, 8.1% vs 0.0%; *p* = 0.010) than CU participants without stroke. None of the patients with stroke and CU participants had CAA markers including cSS and strictly lobar MBs.

Regarding the development of PSCI in patients with stroke, 3 months after the stroke, 12/37 (32.4%) patients developed PSCI (Table 1). The mean age (*p* = 0.238), female ratio (*p* = 0.228), years of education (*p* = 0.128), and rates of hypertension (*p* = 0.168) did not differ between the PSCI and PSNC groups. Patients with PSCI had a higher frequency of diabetes (58.3%) than  those with PSNC (16.0%; *p* = 0.024).

**Table 1:** Demographic and clinical characteristics of the study participants

Group	Patients with stroke				CU without stroke (*n* = 37)	*p*^¥^
	PSNC (*n* = 25)	PSCI (*n* = 12)	p*	Total (*n* = 37)		
*Demographics*						
Age, years	71.3 ± 8.0	74.7 ± 8.0	0.238	72.4 ± 8.1	72.6 ± 8.2	0.920
Sex, female	6 (24.0%)	6 (50.0%)	0.228	12 (32.4%)	13 (35.1%)	1.000
Education, years	9.4 ± 4.2	6.9 ± 5.1	0.128	8.6 ± 4.6	10.0 ± 4.8	0.196
* APOE*, *e4* carrier	6 (24.0%)	7 (58.3%)	0.093	13 (35.1%)	7 (18.9%)	0.116
Hypertension	16 (64.0%)	11 (91.7%)	0.168	27 (73.0%)	17 (45.9%)	0.033
Diabetes	4 (16.0%)	7 (58.3%)	0.024	11 (29.7%)	11 (29.7%)	1.000
*Cognition*						
K-MMSE	26.1 ± 3.8	22.3 ± 3.7	0.008	24.9 ± 4.1	27.9 ± 1.9	< 0.001
CDR-SOB	0.90 ± 0.54	2.71 ± 1.36	0.001	1.49 ± 1.22	0.20 ± 0.36	< 0.001
*Aβ deposition*						
Aβ positivity	4 (16.0%)	7 (58.3%)	0.024	11 (29.7%)	6 (16.2%)	0.167
*cSVD burden*						
WMH severity			0.228			0.010
Moderate WMH	5 (20.0%)	4 (33.3%)		9 (24.3%)	2 (5.4%)	
Severe WMH	1 (4.0%)	2 (16.7%)		3 (8.1%)	0 (0.0%)	
Lacune counts	0.7 ± 1.4	0.7 ± 1.1	0.977	0.7 ± 1.3	0.1 ± 0.3	0.006
MB counts	0.6 ± 1.4	0.6 ± 1.1	0.972	0.6 ± 1.3	0.4 ± 1.2	0.458
*CAA markers*						
cSS	0 (0.0%)	0 (0.0%)		0 (0.0%)	0 (0.0%)	
Strictly lobar MB	0 (0.0%)	0 (0.0%)		0 (0.0%)	0 (0.0%)	

Values are presented as mean ± SD or *n* (%)

*Abbreviations*: *Aβ* Amyloid-β, *APOE* Apolipoprotein E, *CDR-SOB* Clinical Dementia Rating-Sum of Box, *cSVD* Cerebral small vessel disease, *CU* Cognitively unimpaired, *K-MMSE* Korean version of the Mini-Mental Status Examination, *MB* Microbleed, *PSNC* Post-stroke normal cognition, *PSCI* Post-stroke cognitive impairment, *WMH* White matter hyperintensity, *SD* Standard deviation

^*^p-value was obtained by independent *t*-tests and chi-squared tests between the PSNC and PSCI groups

^¥^*p*-value was obtained by independent *t*-tests and chi-squared tests between patients with stroke and CU without stroke groups

### Aβ positivity between stroke and CU without stroke groups

Aβ positivity was higher in the stroke group (29.7%) than in the age- and sex-matched CU without stroke group (16.2%), while the difference was not significant.

### Cognitive trajectory between the stroke and CU without stroke groups

Compared with the CU without stroke group, the stroke group was associated with lower K-MMSE (at 3 months, *β* = − 2.079, *p* = 0.004; at 12 months, *β* = − 2.392, *p* = 0.002) and CDR-SOB scores (at 3 months, *β* = 1.124, *p* < 0.001; at 12 months, *β* = 0.966, *p* = 0.001), irrespective of Aβ positivity and WMH severity. However, the stroke group was not associated with faster deterioration in CDR-SOB (*β* = 0.098, *p* = 0.592) and K-MMSE score (*β* = − 0.676, *p* = 0.076), after controlling for Aβ positivity and WMH severity.

### Aβ deposition and PSCI development in patients with stroke

**Fig. 1:**
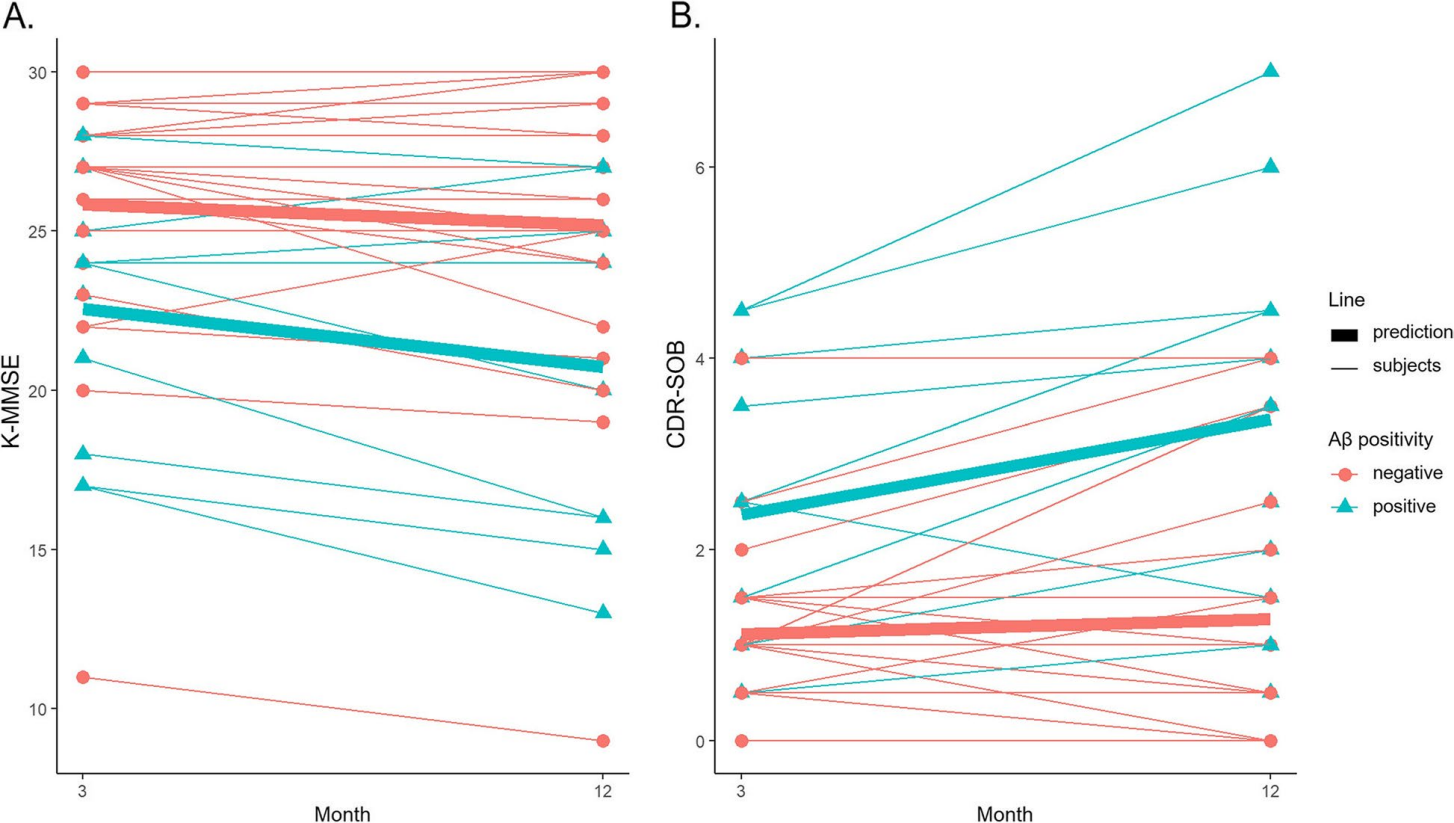
Cognitive trajectory according to Aβ positivity. The *y*-axis represents the K-MMSE score (**a**) or CDR-SOB score (**b**) 3 and 12 months after stroke. Thin and thick lines represent the scores of each patient and the predicted scores, respectively. *Abbreviations*: Aβ, amyloid-β; CDR-SOB, Clinical Dementia Rating-Sum of Box; K-MMSE, Korean version of the Mini-Mental Status Examination

Among patients with small subcortical infarction, Aβ positivity (odds ratio [OR] = 72.2, *p* = 0.024) was associated with PSCI development, independently of WMH (Table 2; Fig. 1). Additionally, the presence of diabetes (OR = 32.9, *p* = 0.042) and severe WMH (OR = 214.0, *p* = 0.046) were associated with PSCI development.

**Table 2:** Association of Aβ positivity with PSCI

		Presence of PSCI in patients with stoke	
		OR (95% CI)^a^	*p*
Risk factors	Hypertension	9.5 (0.6–437.4)	0.161
	Diabetes	32.9 (2.15–2962.9)	0.042
Aβ deposition	Aβ positivity	72.2 (3.87–9355.2)	0.023
WMH	Moderate WMH	3.6 (0.2–100.3)	0.391
	Severe WMH	214.0 (2.8–224,228.2)	0.046

*Abbreviations*: *Aβ* Amyloid-β, *PSCI* Post-stroke cognitive impairment, *OR* Odds ratio, *CI* confidence interval, *WMH* White matter hyperintensity

^*^OR was obtained by logistic regression analysis with hypertension, diabetes, Aβ positivity, and WMH together as predictors and PSCI development as an outcome, after controlling for age, sex, and years of education

### Aβ deposition and cognitive trajectory after stroke

Longitudinal changes in K-MMSE or CDR-SOB scores based on Aβ positivity among patients with small subcortical infarctions are illustrated in Fig. 1. Aβ positivity was associated with faster deterioration in CDR-SOB (*β* = 0.846, *p* = 0.014), while Aβ positivity was not associated with faster decline in K-MMSE score (*β* = − 1.164, *p* = 0.083; Table 3, Fig. 1). Additionally, Aβ positivity was associated with lower K-MMSE (*β* = − 3.380, *p* = 0.016) and CDR-SOB scores (at 3 months, *β* = 1.072, *p* = 0.025; at 12 months, *β* = 1.770, *p* = 0.004), except for K-MMSE score at 3 months (*β* = − 2.363, *p* = 0.071).

**Table 3:** Association of Aβ positivity with cognitive trajectory

	Aβ effect		Time effect		Aβ positivity × time	
	*Β*^a^ (95% CI)	*p*	*Β*^a^ (95% CI)	*p*	*Β*^a^ (95% CI)	*p*
K-MMSE	− 2.218 (− 4.568, 0.133)	0.064	− 0.654 (− 1.375, 0.068)	0.074	− 1.164 (− 2.488, 0.159)	0.083
CDR-SOB	0.998 (0.068, 1.929)	0.036	0.154 (− 0.207, 0.515)	0.393	0.846 (0.185, 1.508)	0.014

*Abbreviations*: *Aβ* Amyloid-β, *CDR-SOB* Clinical Dementia Rating-Sum of Box, *CI* confidence interval, *K-MMSE* Korean version of the Mini-Mental Status Examination

^a^*Β* was obtained by a linear mixed-effect regression analysis with participant as a random effect and Aβ positivity, time, and Aβ positivity × time as fixed effects along with age, sex, education years, hypertension, diabetes, and WMH severity

## Discussion

In the present study, we investigated the relationships between Aβ deposition and the development of PSCI in patients with small subcortical infarctions. Our major findings are as follows: First, Aβ positivity was 29.7% in patients with small subcortical infarctions, which was higher than that in CU participants without stroke. Second, Aβ positivity was predictive of PSCI development regardless of cSVD burden. Finally, Aβ positivity was associated with a poor cognitive trajectory 1 year after stroke. Taken together, our findings suggest that Aβ positivity is an important predictor for PSCI development and cognitive decline over the course of 1 year. Furthermore, considering that AD-related cognitive decline is potentially delayed by pharmacological agents, our results provide evidence that anti-AD medications may be a strategy for preventing cognitive decline in patients with small subcortical infarctions.

Our first major finding was that Aβ positivity was 29.7% in patients with small subcortical infarctions. This frequency was consistent with previous studies, which reported approximately 30% of Aβ positivity in patients with cerebral infarction and SIVD [17–19]. In terms of Aβ positivity in CU participants, our finding that 16.2% of age- and sex-matched CU participants without stroke had Aβ positivity was generally consistent with previous studies, which have shown that the range of Aβ positivity in CU Asians is 15~20% and CU Europeans is 30~40% [5, 20–24]. A recent study has revealed that Aβ positivity in CU Asians (17.5% [30/171]) is lower than that in CU Europeans (30.4% [1250/3947], *p* = 0.002) [20]. Considering the lower frequency of Aβ positivity in CU Asians than in CU Europeans [5, 20–24], Aβ positivity in patients with small subcortical infarctions might be higher than that in normal elderly individuals. Several mechanisms have been proposed to explain the relationship between small subcortical infarctions and Aβ deposition. Specifically, cessation of cerebral blood flow induces rapid Aβ deposition by decreasing the activity of Aβ degradation enzymes, such as neprilysin [4, 25]. Alternatively, patients with small subcortical infarctions may have a predisposition to cSVD, which is closely related to Aβ deposition. Several autopsy studies have demonstrated an overlap between Aβ and cSVD burdens [26, 27]. The interaction between Aβ and cSVD may be explained by the possibility that cSVD hampers Aβ clearance [28, 29].

We also found that patients with small subcortical infarctions had a higher frequency of hypertension and WMH burdens than CU participants without stroke. Considering the relationship between small subcortical infarction and cSVD, this finding was what we expected. Hypertension is the most important modifiable risk factor for cSVD [30].

Our second major finding was that Aβ positivity was predictive of PSCI development regardless of the cSVD burden, suggesting that coexistent AD pathology may play a vital role in the development of PSCI after stroke. This finding is comparable to previous findings that Aβ positivity is an important factor in PSCI development [18, 31, 32]. Recent studies have found that plasma AD biomarkers are associated with the development of PSCI. However, several previous studies have shown that Aβ positivity is not associated with PSCI development [33–35]. This discrepancy might be explained by the differences in the inclusion criteria of the study participants (small subcortical infarctions without strategic lesions in the current study versus all etiologies of stroke or cerebral infarctions in the previous study). Patients with territorial infarction are more likely to have language problems and physical disabilities, which in turn lead to cognitive decline regardless of Aβ positivity. Additionally, patients with strategic infarctions are prone to cognitive impairment due to the infarcted lesion itself. Thus, these factors may override the effect of Aβ in a large proportion of stroke patients.

Although further studies are required to elucidate the mechanism underlying the relationship between Aβ positivity and PSCI, neuroinflammation may mediate this association. Neuroinflammation is a common trigger for neuronal damage in individuals with Aβ and cerebral infarctions. It is well known that Aβ deposition in the brain starts to accumulate 10 years before the onset of clinical symptoms. After the deposition of Aβ, neuroinflammation may lead to neurodegeneration, a surrogate marker of cognitive decline, even in the non-demented stage [36]. Specifically, microglial activation can switch from an anti-inflammatory phenotype (M2) to a pro-inflammatory phenotype (M1) during AD. Cerebral ischemia-related excitotoxicity may also contribute to the activation of the inflammatory response, eventually resulting in neuronal damage. Thus, individuals with Aβ deposition may be more vulnerable to cerebral infarction-induced neuroinflammation, which leads to neurodegeneration. Alternatively, cerebral ischemia may aggravate the Aβ-related neuroinflammation.

We also found that pre-existing WMH and diabetes were predictive of PSCI development. Although the association between pre-existing WMH and PSCI development was expected, this was again replicated in our groups, a finding suggestive of the importance of pre-existing WMH on cognitive impairment after small subcortical infarctions [37–39]. Regarding diabetes, this finding is consistent with those of previous studies [40, 41]. Diabetes represents a metabolically unhealthy condition and has been found to be associated with inflammation, which may aggravate the neuronal damage after stroke [42].

Our final major finding was that Aβ positivity was associated with faster deterioration in CDR-SOB for 1 year after stroke. In patients with small subcortical infarctions, the relationship between Aβ positivity and long-term cognitive trajectory remains controversial. Our findings suggest that Aβ positivity is associated with delayed-onset PSCI. Recent studies using plasma AD biomarkers reported comparable results [32, 43]. Aβ positivity is an important predictor of SVID development and exerts a synergistic effect on cognitive decline with cSVD burden in patients with severe WMH burden, which is another form of cSVD [44]. However, Aβ positivity was not associated with a rapid decline in the K-MMSE scores. This may be explained by the characteristics of the PSCI and the intrinsic limitations of the K-MMSE. Because patients with PSCI are more vulnerable to cognitive decline in frontal-executive function than in other cognitive domains, the K-MMSE may have underestimated cognitive decline in the present study.

### Limitations

In the present study, we investigated the association of Aβ deposition on PET with the development of PSCI and long-term prognosis in patients with small subcortical infarction. However, our study had several limitations that need to be addressed. First, the sample size is relatively small. Second, we could not assess long-term cognitive trajectories using a detailed neuropsychological battery. However, this is mitigated to a certain extent by the fact that the CDR-SOB score is a well-validated outcome measure that is widely used in clinical trials of AD and PSCI. Third, the generalizability of the present study to all patients with stroke should be treated with caution because only participants with small subcortical infarctions were enrolled. Nevertheless, our study provides a comprehensive understanding of the relationship between Aβ positivity and cognitive impairment in patients with small subcortical infarctions.

## Conclusions

In conclusion, Aβ positivity is an important predictor for the development of PSCI. Furthermore, preclinical AD pathology was predictive of a poor cognitive trajectory. Therefore, anti-AD medications may contribute to delayed cognitive decline in patients with small subcortical infarctions, although it is necessary to select the patient carefully with consideration for the MBs count and WMH severity.

## Data Availability

The anonymized data for the analyses presented in this report are available upon request from the corresponding authors.

## Abbreviations

AD: Alzheimer’s disease
APOE: Apolipoprotein E
Aβ: Amyloid-β
BAPL: Brain amyloid-plaque load
CDR-SOB: Clinical Dementia Rating-Sum of Box
CI: Confidence interval
COWAT: Controlled Oral Word Association Test
cSVD: Cerebral small vessel disease
CT: Computed tomography
CU: Cognitively unimpaired
DWMH: Deep white matter hyperintensities
FLAIR: Fluid-attenuated inversion recovery
IQCODE: Informant Questionnaire on Cognitive Decline in the Elderly
K-BNT: Korean version of the Boston Naming Test
K-MMSE: Korean version of the Mini-Mental Status Examination
K-VCIHS-NP: Korean version of the Vascular Cognitive Impairment Harmonization Standards neuropsychological battery
MB: Microbleed
MRI: Magnetic resonance imaging
NIHSS: National Institutes of Health Stroke Scale
OR: Odds ratio
PET: Positron emission tomography
PSCI: Post-stroke cognitive impairment
PSNC: Post-stroke normal cognition
PWMH: Periventricular white matter hyperintensities
RCFT: Rey Complex Figure Test
RCTU: Regional cortical tracer uptake
SD: Standard deviation
SIVD: Subcortical ischemic vascular dementia
SVLT: Seoul Verbal Learning Test
WMH: White matter hyperintensity(ies)
